# Morphological, Cytological and Molecular Studies and Feeding and Defecation Pattern of Hybrids from Experimental Crosses between *Triatoma sordida* and *T. rosai* (Hemiptera, Triatominae)

**DOI:** 10.3390/pathogens11111302

**Published:** 2022-11-06

**Authors:** Roberto Dezan Vicente, Fernanda Fernandez Madeira, Kelly Cristine Borsatto, Ariane Cristina Caris Garcia, Daniel Cesaretto Cristal, Luiza Maria Grzyb Delgado, Isadora de Freitas Bittinelli, Denis Vinicius De Mello, Yago Visinho Dos Reis, Amanda Ravazi, Cleber Galvão, Maria Tercília Vilela De Azeredo-Oliveira, João Aristeu Da Rosa, Jader De Oliveira, Kaio Cesar Chaboli Alevi

**Affiliations:** 1 Instituto de Biociências de Botucatu, Universidade Estadual Paulista “Júlio de Mesquita Filho” (UNESP), Botucatu 18618-689, SP, Brazil; 2 Instituto de Biociências, Letras e Ciêcias Exatas, Universidade Estadual Paulista “Júlio de Mesquita Filho” (UNESP), São José do Rio Preto 15054-000, SP, Brazil; 3 Laboratório Nacional e Internacional de Referência em Taxonomia de Triatomíneos, Instituto Oswaldo Cruz (FIOCRUZ), Rio de Janeiro 21040-360, RJ, Brazil; 4 Laboratório de Parasitologia, Faculdade de Ciências Farmacêuticas, Universidade Estadual Paulista “Júlio de Mesquita Filho” (UNESP), Araraquara 14801-902, SP, Brazil; 5 Laboratório de Entomologia em Saúde Pública, Faculdade de Saúde Pública, Universidade de São Paulo (USP), São Paulo 01246-904, SP, Brazil

**Keywords:** Chagas disease vectors, hybridization, molecular biology, morphology, cytogenetics

## Abstract

Under laboratory conditions, *Triatoma rosai* and *T. sordida* are able to cross and produce hybrids. In the face of climate and environmental changes, the study of hybrids of triatomines has evolutionary and epidemiological implications. Therefore, we performed morphological, cytological and molecular studies and characterized the feeding and defecation pattern of hybrids from crosses between *T. sordida* and *T. rosai*. The morphological characterization of the female genitalia of the hybrids showed that characteristics of both parental species segregated in the hybrids. Cytogenetic analyzes of hybrids showed regular metaphases. According to molecular studies, the mitochondrial marker Cytochrome B (*CytB*) related the hybrids with *T. sordida* and the nuclear marker Internal Transcribed Spacer 1 (ITS-1) related the hybrids with *T. rosai*. Both parents and hybrids defecated during the blood meal. Thus, the hybrids resulting from the cross between *T. sordida* and *T. rosai* presented segregation of phenotypic characters of both parental species, 100% homeology between homeologous chromosomes, phylogenetic relationship with *T sordida* and with *T. rosai* (with *CytB* and ITS-1, respectively), and, finally, feeding and defecation patterns similar to the parents.

## 1. Introduction

Chagas disease is a neglected disease caused by the protozoan *Trypanosoma cruzi* (Chagas, 1909) (Kinetoplastida, Trypanosomatidae) [1,2] that affects about seven million people worldwide [1,2]. This disease is mostly transmitted when humans come into contact with faeces and/or urine of triatomines (Hemiptera, Triatominae) infected by *T. cruzi* (vector-borne transmission) [1,2]. As Chagas disease has no cure in the chronic phase and the acute phase is usually asymptomatic [1,2], the World Health Organization points out that vector control is considered as the main measure to reduce the incidence of new infections [1,2].

There are currently 157 species (154 extant species and three fossils) grouped into 18 genera and five tribes that are potential vectors of *T. cruzi* [3,4,5]. In the last ten years (2012–2022), 13 species of triatomines were described [3,5]. However, among them, only two show clues of house invasion or domiciliation: *Triatoma huehuetenanguensis* Lima-Cordón et al., 2019 (Hemiptera, Triatominae) and *T. rosai* Alevi et al., 2020 [6].

*Triatoma rosai* is a related species of *T. sordida* (Stål, 1859), and was recently described using integrative taxonomy [7]. Although phylogenetically related, these species show differences in morphological, morphometric, genetic, and cytogenetic aspects, as well as in electrophoresis and cuticular hydrocarbons pattern [7]. Under laboratory conditions, these species are able to cross and produce hybrids (although the vast majority of hybrid offspring die before reaching adulthood) [7].

The study of hybridization capacity is an important taxonomic tool for Triatominae [7,8,9,10], because the characterization of pre- and/or post-zygotic reproductive barriers allows confirming the specific status of parental species from the biological species concept [11,12,13]. Furthermore, in the face of anthropogenic climate and environmental changes that are producing significant changes in the distribution pattern, natural history and behavior of species (including pathogens and disease vectors) [14,15], the study of hybrids of these insect vectors has evolutionary and, above all, epidemiological implications.

Shorter defecation time [16] and greater fitness [17,18] has been observed in the hybrids resulting from crosses between *Triatoma* spp., demonstrating that that triatomine hybrids can play an important role in the transmission of Chagas disease [16,17,18,19,20]. Both *T. rosai* and *T. sordida* are species that have already been collected naturally infected by *T. cruzi* [21,22,23,24,25] and that have vector importance for the epidemiology of Chagas disease.

Based on the above, we performed morphological, cytological and molecular studies and we characterized the feeding and defecation pattern of hybrids from experimental crosses between *T. sordida* and *T. rosai*.

## 2. Materials and Methods

### 2.1. Sampling

We examined specimens of *T. rosai* from Department San Miguel, Province of Corrientes, Argentina, specimens of *T. sordida* from Seabra, Bahia, Brazil and adult hybrids resulting from the cross between *T. rosai* ♀ and *T. sordida* ♂ and between *T. rosai* ♂ and *T. sordida* ♀. The analyzed species came from live colonies kept in the Triatominae Insectarium of the São Paulo State University “Julio de Mesquita Filho”, School of Pharmaceutical Sciences, Araraquara, São Paulo, Brazil. In addition, interspecific crosses were also carried out in the Insectarium to obtain hybrids in both gender combinations (as detailed by Alevi et al. [7]).

### 2.2. Morphological Studies in Scanning Electron Microscopy

For morphological characterization of the triatomines in Scanning Electron Microscope (SEM) (Topcon, Hasunuma-cho, Itabashi-Ku, Tokyo, Japan) (according to Rosa et al. [26]), four individuals of *T. rosai*, *T. sordida* and hybrids from both directions of crosses were used, emphasizing the study of the female external genitalia. For this study, the insects were cleaned in ultrasonic devices, dehydrated in graded series of alcohol, oven-dried at 45 °C for 20 min, and then fixed in small aluminum cylinders with colorless enamel. Afterward, they were metalized by sputtering for two minutes with 10 mA of power. After the metallization process, the samples were analyzed and photographed on the Topcon SM-300 SEM (Digital, Hasunuma-cho, Tokyo, Japan).

### 2.3. Cytogenetic Analysis

Four adult male hybrids from each gender combination were dissected and their testes removed and stored in a methanol:acetic acid solution (3:1). Slides were prepared by the cell-crushing technique (as described by Alevi et al. [27]), and cytogenetic analyses were performed to characterize spermatogenesis, with emphasis on the degree of pairing between the homeologous chromosomes, using the lacto-acetic orcein technique [27,28]. The slides were examined under a light microscope (Jenamed; Carl Zeiss, Jena, Germany) that was coupled with a digital camera with a 1000-fold magnification; AxioVision LE version 4.8 imaging software (Carl Zeiss) was used for analysis.

### 2.4. Molecular Analysis

Sequences of two molecular markers [Cytochrome B (*CytB*) and Internal Transcribed Spacer 1 (ITS-1)] obtained from *T. sordida* (*n* = 4), *T. rosai* (*n* = 4) and their hybrids (*n* = 4) as well as from *T. infestans* (Klug, 1834) (placed as outgroup) (Table 1) were submitted to the MEGA X program [29] and aligned by the Muscle method [30]. The alignments were concatenated by name using the Seaview4 program [31] and converted with the Mesquite program [32] for analysis in MrBayes 3.2 [33]. The data of each marker was also converted individually for analysis.

The best nucleotide substitution model (lowest Akaike Information Criterion value) for each marker was determined using the jModelTest 2 program [30], being HKY +G for *CytB* and GTR for ITS-1.

The phylogenetic reconstruction by Bayesian approach was performed in MrBayes 3.2 [34] for each marker, with a total of 100 million generations. Trees were sampled every 1000 generations in two independent runs, with burn-in set to 25%. The Tracer v. 1.7 program [35] was used to verify the stabilization (ESS values above 200) of the sampled trees and the generated phylogenetic tree of each analysis was viewed and edited in the FigTree v.1.4.4 [35] program, being rooted at the midpoint.

### 2.5. Feeding and Defecation Behavior

**Figure 1:**
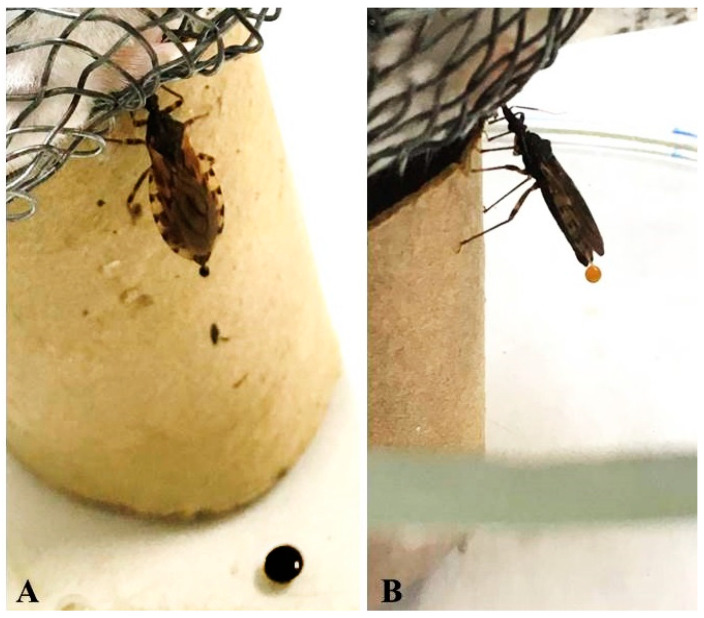
Hybrids defecating (**A**) and urinating (**B**) during blood feeding.

The feeding and defecation dynamics of *T. rosai*, *T. sordida*, and experimental hybrids were evaluated based on Diotaiuti et al. [36] with modifications: 20 adults of each species/hybrid were fed with mice and the mean period of time for feeding and mean period of time after beginning of feeding until defecation were monitored individually for one hour. The determination of the period of feeding time started with the beginning of the feeding process (when the insect inserted the mouthparts into the mouse) and ended when the insect stopped performing blood ingestion (when removing the mouthparts out of the mouse). The determination of the period of time until defecation started with the beginning of the feeding process and ended with the first release of excreta (feces/urine) by the insect (Figure 1). The period of feeding time and of the period of time until defecation were compared between hybrids and each parental species using ANOVA. Data between males and females (without distinction of species/hybrids) were also compared using Student’s t-test. The results were considered to be statistically significant when *p* ≤ 0.05. Analyzes were conducted in Jasp 0.16.2 [37]. All animal experiments were conducted in accordance with the Guidelines for the Treatment of Experimental Animals according to the ethical issues approved by the Ethics Committee for Animal Use of the FCFAR/UNESP, Brazil (CEUA/FCF/CAr n° 18/2019) and the National Council for Animal Experiment Control of the FCFAR/UNESP, Brazil (CIAEP/CONCEA n° 02.0082.2019).

## 3. Results and Discussion

**Figure 2:**
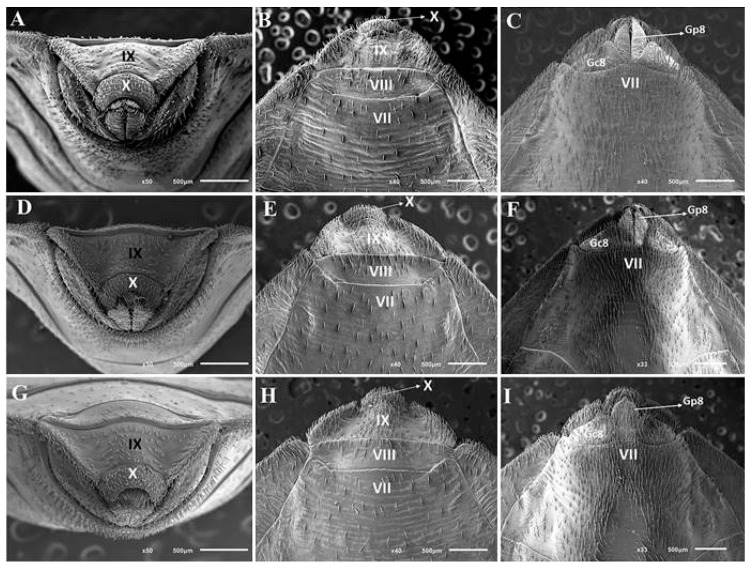
External female genitalia of *T. rosai* (**A**–**C**) from hybrids resulting from the cross between *T. rosai* ♀ and *T. sordida* ♂ (**D**–**F**) and *T. sordida* (**G**–**I**). Gc8: Gonocoxite VIII; Gp8: gonapophysis VIII; IX, VII and IX: sternites and X: segment.

Morphological [8,38,39,40], morphometric [38], genetic [39], cytogenetic [8,9,38,41], molecular [39], behavioral [42], and epidemiological [16,20] aspects have already been studied in hybrids of Chagas disease vectors. The morphological characterization of the female genitalia of the hybrids resulting from the cross between *T. rosai* ♀ and *T. sordida* ♂ showed that in dorsal view (Figure 2A,D,G), *T. rosai* pattern (tenth segment form) and *T. sordida* pattern (ninth segment central form and eighth segment form) was observed; in posterior view (Figure 2B,E,H), *T. sordida* pattern (central portion of the ninth segment) and intermediate pattern (shape and length of the tenth segment) was notified, and in ventral view (Figure 2C,F,I), only the *T. sordida* pattern (line that divides the seventh and eighth gonocoxites segment and gonapophysis and shape of the eighth gonocoxites) was segregated.

**Figure 3:**
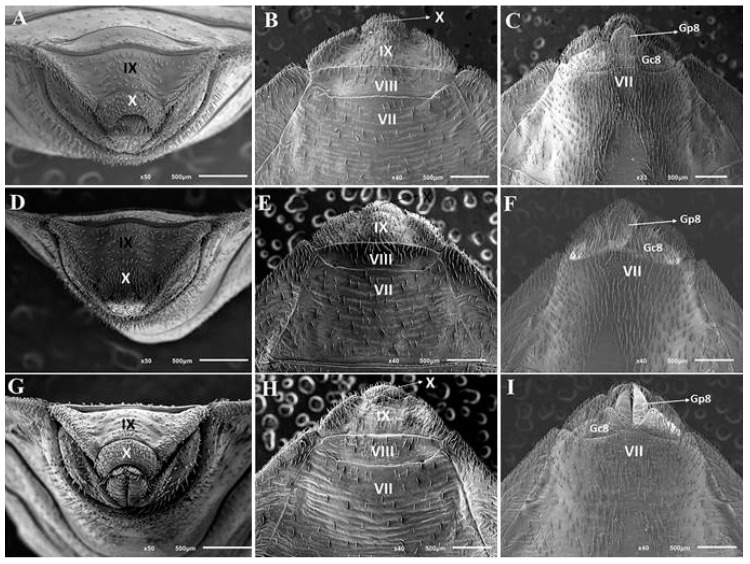
External female genitalia of *T. rosai* (**A**–**C**) from hybrids resulting from the cross between *T. sordida* ♀ and *T. rosai* ♂ (**D**–**F**) and *T. sordida* (**G**–**I**). Gc8: Gonocoxite VIII; Gp8: gonapophysis VIII; IX, VII and IX: sternites and X: segment.

The morphological characterization of the female genitalia of the hybrids resulting from the cross between *T. sordida* ♀ and *T. rosai* ♂ showed that in dorsal view (Figure 3A,D,G), *T. sordida* pattern (central form of the ninth segment and form of the tenth segment) and *T. rosai* pattern (form of the eighth segment) were observed; in posterior view (Figure 3B,E,F), only *T. sordida* pattern (central portion of the ninth segment and shape and length of the tenth segment) was notified, and in ventral view (Figure 3C,F,I), only *T. rosai* pattern (line dividing the seventh segment and the eighth gonocoxites and gonapophysis and form of the eighth gonocoxites) was segregated.

The study of the segregation of phenotypic characteristics in Triatominae has been carried out for over 50 years [43]. Both segregation patterns similar to those observed for hybrids of *T. sordida* and *T. rosai*, as well as divergent patterns were characterized in the genus *Triatoma* Laporte, 1832: hybrids resulting from the crosses between *T. b. brasiliensis* Neiva, 1911 ♀ x *T. lenti* Sherlock & Serafim, 1967 ♂, *T. juazeirensis* Costa & Felix (2007) ♀ x *T. lenti* ♂, and *T. melanica* Neiva & Lent, 1941 ♀ x *T. lenti* ♂ showed segregation of characteristics of both parental species [40], hybrids resulting from the cross between *T. lenti* x *T. sherlocki* Papa et al. (2002) and between *T. juazeirensis* x *T. sherlocki* showed intermediate characteristics [38,42], hybrids resulting from the crosses between *T. lenti* ♀ x *T. juazeirensis* ♂, *T. b. macromelasoma* Galvão, 1956 ♀ x *T. lenti* ♂, *T. lenti* ♀ x *T. melanica* ♂, and *T. infestans* and *T. rubrovaria* (Blanchard, 1843) showed a specific pattern of *T. lenti*, *T. lenti*, *T. melanica*, and *T. rubrovaria*, respectively [40,43].

Morphological studies on hybrids have taxonomic, evolutionary and epidemiological importance [7,8,9,10,38,39,40,41,42,43,44,45]. Recently, Pinotti et al. [40] analyzed the phenotypic segregation in hybrids of *T. brasiliensis* subcomplex and, based on the observation of different patterns (intermediate, of both parents or just one parent), they highlighted the importance of integrative taxonomy for the correct identification of Chagas disease vectors grouped in the subcomplex if natural hybridization events occur. In addition, in the studies presented by Almeida et al. [42] who crossed the brachypterous *T. sherlocki* with the macropterous *T. juazeirensis*, the hybrids presented intermediate patterns, which provided greater fitness than the parents in the home invasion process (since they can do this either walking or flying).

**Figure 4:**
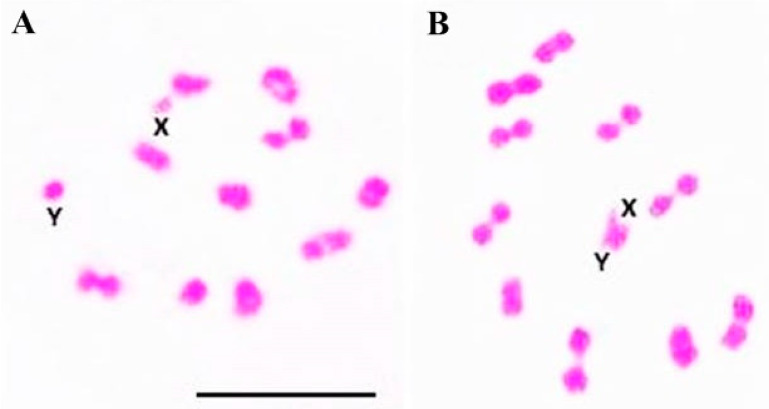
Metaphases I of hybrids resulting from crosses between *T. sordida* ♀ and *T. rosai* ♂ (**A**) and *T. sordida* ♂ and *T. rosai* ♀ (**B**). Note 100% pairing between homeologous chromosomes. X: X sex chromosome, Y: Y sex chromosome. Bar: 10 μm.

Cytogenetic analyzes of *T. sordida* and *T. rosai* hybrids (both gender combinations) showed regular metaphases, with 100% pairing between the homologous chromosomes (Figure 4A,B). In general, phylogenetically related species show a higher degree of homeology between chromosomes in metaphase I [46]. This can be observed, for example, for the hybrids of the species of the monophyletic *T. brasilieinsis* subcomplex [41]. Although the post-zygotic barrier characterized for the cross between *T. sordida* and *T. rosai* is the infeasibility of the hybrid [7], the reproductive barrier characterized among the species of the *T. brasiliensis* subcomplex is the hybrid collapse [38]. This event was characterized by chromosome pairing errors observed in second-generation hybrids (F2), which resulted in the formation of nonviable gametes [8,9,10,11,12,13].

**Figure 5:**
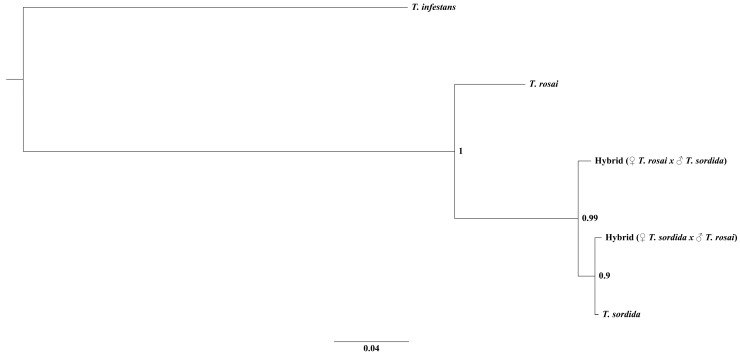
Phylogenetic relationship between *T. rosai*, *T. sordida*, and experimental hybrids with the *CytB* gene. The numbers in the nodes indicates the posterior probability.

**Figure 6:**
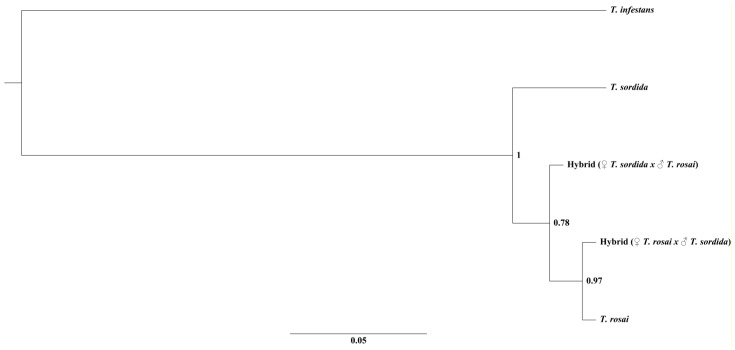
Phylogenetic relationship between *T. rosai*, *T. sordida*, and experimental hybrids with the ITS-1 molecular marker. The numbers in the nodes indicates the posterior probability.

There is only one molecular study on triatomine hybrids, in which the authors analyzed the relationship between *T. longipennis* Usinger 1939, *T. pallidipennis* Stal, 1872, *T. picturata* Usinger 1939 and their experimental hybrids through the Cytochrome C Oxidase Subunit I (*COI*) gene [39]. We performed molecular studies with the *CytB* (Figure 5) and ITS-1 (Figure 6) molecular markers in *T. sordida*, *T. rosai* and in the experimental hybrids: the mitochondrial marker related the hybrids with *T. sordida* (Figure 5) and the nuclear marker related the hybrids with *T. rosai* (Figure 6).

Mitochondrial genes are maternally inherited [47], so it was expected that in the resulting phylogeny of *CytB* the hybrids would group together with the respective female species used in the cross (Figure 5). However, as mentioned above, both hybrids clustered with *T. sordida*. The knowledge of gene segregation in triatomine hybrids is still uncertain, as Davila-Barboza et al. [39], when analyzing hybrids resulting from the cross between *T. picturata* ♀ and *T. pallidipennis* ♂ for the *COI* gene, observed that these organisms were not directly related to the parental species, but with *T. longipennis* and with hybrids resulting from the cross between *T. longipennis* ♀ and *T. pallidipennis* ♂ and between *T. longipennis* ♀ and *T. picturata* ♂. On the other hand, nuclear genes show genetic recombination [48,49], which justifies the randomness of the hybrids in the phylogeny. However, with the analysis of ITS-1, both hybrids were closer to *T. rosai* (Figure 6), demonstrating that there was probably a dominance of segregation of the genotypic characteristics of this parental species in the hybrids.

The feeding and defecation pattern of *T. rosai*, *T. sordida* and the experimental hybrids was evaluated (Table 2). Both parents and hybrids defecated during the blood meal (Table 2), however, there was no significant difference between the times of feeding and defecation of the hybrids in relation to the parents (*p* = 0.595 and *p* = 0.544, respectively). Despite this, we could observe a significant difference in feeding (*p* = 0.005) and defecation (0.009) times between males and females (grouping data for each species and hybrids), the shortest times being observed for females. These results are important from an epidemiological point of view, as a good vector of Chagas disease, in general, has a shorter period of time between the beginning of blood ingestion and first defecation, depositing *T. cruzi* while still feeding [50].

The time interval before beginning of feeding, for feeding, and until defecation for *T. mazzottii* Usinger, 1941, *T. pallidipennis*, and *T. phyllosomus* Burmeister, 1835 and their laboratory hybrids, as well as *T. pallidipennis*, *T. longipennis*, *T. picturata*, and their laboratory hybrids were evaluated [16,17,18,19,20]. According to these data, the hybrid cohorts were more effective vectors of *T. cruzi* than their parental species. In the same way, López et al. [51] analyzed the vector competence of hybrids resulting from the cross between *T. infestans* and *T. platensis* Neiva, 1913 and, based on the blood ingestion velocity, the amount of blood ingested, and the short time required for the production of the first defecation, the hybrid can be considered as a competent *T. cruzi* vector.

## 4. Conclusions

Based on the above, the hybrids resulting from the cross between *T. sordida* and *T. rosai* presented segregation of phenotypic characters of both parental species, 100% homeology between metaphase chromosomes, phylogenetic relationship with *T sordida* (with the *CytB* gene) and with *T. rosai* (with the ITS-1 molecular marker) and, finally, feeding and defecation patterns similar to the parents, highlighting the possible vector competence of these insects for Chagas disease (because they defecate during a blood meal).

## Figures and Tables

**Table 1:** Species and molecular markers used in the phylogenetic studies.

Species	*Cyt B*	ITS-1
*T. sordida*	MH054940	*
*T. rosai*	*	*
Hybrid ^1^	*	*
Hybrid ^2^	*	*

* Sequences obtained in this study ^1^ resulting from the cross between *T. sordida* ♂ and *T. rosai* ♀; ^2^ resulting from the cross between *T. sordida* ♀ and *T. rosai* ♂.

**Table 2:** Mean period of time for feeding and mean period of time after beginning of feeding until defecation (*n* = 20 in each group).

	Feeding	Defecation
*T. sordida* ♀	30:29	18:47
*T. sordida* ♂	32:56	23:09
*T. rosai* ♀	31:49	22:02
*T. rosai* ♂	34:27	25:11
Hybrid ^1^ ♀	32:00	19:14
Hybrid ^1^ ♂	35:46	24:18
Hybrid ^2^ ♀	31:17	21:01
Hybrid ^2^ ♂	36:12	23:15

^1^ Resulting from the cross between *T. sordida* ♂ and *T. rosai* ♀; ^2^ resulting from the cross between *T. sordida* ♀ and *T. rosai* ♂.

## Data Availability

All relevant data are within the manuscript.
